# The Impact of Starches from Various Botanical Origins on the Functional and Mechanical Properties of Anhydrous Lotion Body Bars

**DOI:** 10.3390/polym17131731

**Published:** 2025-06-21

**Authors:** Agnieszka Kulawik-Pióro, Beata Fryźlewicz-Kozak, Iwona Tworzydło, Joanna Kruk, Anna Ptaszek

**Affiliations:** 1 Department of Organic Chemistry and Technology, Faculty of Chemical Engineering and Technology, Cracow University of Technology, Warszawska 24, 31-155 Cracow, Poland; 2 Department of Engineering and Machinery for Food Industry, Faculty of Food Technology, University of Agriculture in Krakow, Balicka 122, 30-149 Krakow, Poland; joanna.kruk@urk.edu.pl; 3 Department of Chemical and Process Engineering, Faculty of Chemical Engineering and Technology, Cracow University of Technology, Warszawska 24, 31-155 Cracow, Poland; beata.fryzlewicz-kozak@pk.edu.pl; 4 Centre for Innovation and Research on Prohealthy and Safe Food, University of Agriculture in Krakow, Balicka 104, 30-149 Krakow, Poland; anna.ptaszek@urk.edu.pl

**Keywords:** starch, lotion body bars, rheological properties, mechanical properties

## Abstract

Starch, as a natural, low-cost, and vegan-friendly raw material, aligns well with the growing demand for sustainable, zero-waste, and waterless cosmetic products. Its biodegradability and natural origin allow for minimal environmental impact during production and disposal. Anhydrous lotion body bars, solid and water-free alternatives to traditional moisturizers, offer high concentrations of active ingredients that are more effective and have a longer shelf life. Their solid form enables packaging in paper-based containers, reducing plastic waste. To address formulation challenges such as excessive greasiness, poor absorption, or lack of structural stability, which are often associated with the high oil content of anhydrous body lotion bars, starch may serve as a promising natural additive. The aim of this study was to optimize the formulation of an innovative starch-based anhydrous lotion bar. For this purpose, physicochemical analyses of starches from various botanical sources (corn, rice, tapioca, waxy corn and potato) were performed, along with evaluations of the functional (including commercially acceptable form, hardness sufficient for application, product stability, reduced greasiness and stickiness) and mechanical properties of the resulting bars. Additionally, the rheological behavior was described using the De Kee model. The results indicate that a 2.5% starch addition, regardless of its botanical origin, provides the best balance between viscosity and ease of application. Moreover, starches with a low moisture content and high oil absorption capacity effectively reduce the greasy skin sensation. These findings demonstrate the potential of starch as a natural multifunctional additive in the development of stable, user-friendly anhydrous lotion body bars.

## 1. Introduction

Starches of natural origin play a significant role in the food, pharmaceutical and cosmetic industries [1,2], serving as biodegradable raw materials with multifunctional properties. The most commonly used starches in cosmetics include potato, rice, corn, oat, and tapioca starch. Recent studies have explored the use of alternative plant sources for starch extraction, including bananas [1], pinhão seed [3], or Guapo (*Myrosma cannifolia*) [4]. In cosmetic products, starches are used in various forms: native, physically modified (e.g., gelatinized), and chemically modified [1,5].

For years, starches have been proven to be outstanding rheology modifiers, gelling agents and texturizers [1,3,6,7]. In emulsions, they naturally enhance quality and stability [8] and also improve features such as spreadability, oil absorption, film-forming capacity, and heat resistance [4,9,10].

Native starch is widely used in powder-based (solid) products, including face powders, blushes, mattifying fluids, body and foot powders, eye shadows, dry shampoos, baby powders, and even surgical glove powders [1]. It also serves as a natural alternative to talc [1]. In such products, starch effectively absorbs sebum and moisture, prevents caking, and improves adhesion and coverage on the skin—reducing excessive flaking of the cosmetic. In cosmetics products for children, starch provides soothing and anti-irritant effects.

Starch can be also used in sunscreens as an SPF enhancer, especially in combination with inorganic filters [9]. It improves product compressibility and gives the skin a youthful, smooth, and soft appearance [4]. In deodorants, starch acts as a thickening agent and helps control perspiration [7]. Its absorbent nature reduces the greasiness and stickiness of cosmetic products, contributing to a lighter skin feel.

Additionally, starch is incorporated into the formulas of bath salts and effervescent bath bombs, where it serves as a filler. Starch can be a source of minerals and B-group vitamins, and when ground to various particle sizes also exhibits exfoliating properties. Furthermore, starches can act as carriers of essential oils [11] and are compatible with a wide range of cosmetics raw materials. Modified starches, such as hydrolyzed corn starch, hydrolyzed wheat starch and sodium carboxymethyl starch, are commonly used in cleansing products such as body washes [6].

Starch, as a natural, low-cost, and vegan-friendly raw material, aligns well with the current market trend toward the development of natural cosmetics and sustainable packaging. To reduce plastic production, body care products such as balms, lotions, body butters, mousses, and shampoos are increasingly being formulated in solid forms—so-called cubes, bars, or sticks. This physicochemical form enables the use of eco-friendly packaging, such as glass or paper-based containers (which may be made from recycled materials and/or be made biodegradable and compostable).

Solid body butters and lotions (body bars) provide moisturizing, lubricating, regenerating, nourishing, and protective effects. Some formulations also claim slimming properties.

Many body lotion bars available on the market are anhydrous systems. The absence of water in their formulas allows for a higher concentration of active ingredients and results in a more compact product that is easier to store and apply. These products also exhibit longer shelf life, improved physicochemical stability, and reduced microbiological risk. Body bars are often enriched with essential oils, plant extracts, and vitamins. Composed entirely of natural ingredients, they align with zero-waste and waterless beauty philosophies [12,13,14].

Unlike traditional moisturizing balms (lotions) that are dispensed from containers, body bars are applied by directly rubbing the solid product on the skin. Upon contact with the skin, they liquefy due to the user’s body temperature.

Until now, the use of starch in cosmetics has been explored in various formulations, including emulsions intended for sun protection [9], face and body powders [1,15], gels, emulgels [3], and even in nanocapsules derived from pregelatinized modified starch [16]. Relatively recent studies conducted by Ogorzełek et al. [12] focused on the use of starch as a natural exfoliant in scrub bars. However, most applications focus on water-based formulations, and little attention has been paid to its role in anhydrous systems. To the best of our knowledge, there are no existing studies on the application of starch in anhydrous body lotion bars [13,17,18].

From a physical point of view, anhydrous lotion bars are classified as multi-phase systems of a suspension nature, in which the dispersed phase is a solid in loose form and the continuous phase is a mixture of butters and selected vegetable oils. Due to the temperature-dependent properties of the continuous phase, it is necessary to prevent unfavorable phenomena such as phase separation or liquefaction of the continuous phase. Anhydrous body lotion bars often suffer from excessive greasiness, poor absorption, or lack of structural stability. Formulating a stable, pleasant-to-use bar without synthetic ingredients presents a challenge. In this context, starch may serve as a promising natural additive to improve consistency, reduce the stickiness and thickness of the layer left on the skin, and enhance the structural integrity of the product. Additionally, the integration of starch into anhydrous formulation poses specific challenges including sedimentation or incompatibility with a lipid phase.

The aim of the presented study was to optimize the formulation of an innovative starch-based lotion body bar that fits within the framework of natural (produced without using synthetic emulsifiers), waterless, and zero-waste cosmetic trends. The optimization was carried out based on criteria reflecting key functional parameters of the final products, such as a commercially acceptable bar form, hardness sufficient to ensure ease of application, product stability, and reduced greasiness and stickiness after use. For this purpose, physicochemical analyses of starches from various botanical sources were performed, along with evaluations of the functional, mechanical, rheological, and textural properties of the final formulations. The product was designed to contain a high proportion of a lipid phase (cocoa butter) in the composition, while maintaining stability and desirable characteristics and ultimately to be used as a lotion body bar product. The starches used in the study included potato, corn, waxy maize, rice, and tapioca.

## 2. Materials and Methods

### 2.1. Materials

In research, the following commercially available ingredients were used: cocoa butter (INCI: Theobroma Cacao (Cocoa) Seed Butter, Ecospa company, Warsaw, Poland), sunflower oil (INCI: Helianthus Annus Seed Oil, EOL Polska Sp. z o.o, Szamotuły, Poland), beeswax (INCI: Cera Alba, Zrób Sobie Krem, Prochowice, Poland). In this study, Vitamin E was used in the form of tocopheryl acetate (INCI: Tocopheryl Acetate, Zrób Sobie Krem, Prochowice, Poland), which is a stable ester of natural tocopherol.

The starches used in the formulations included: potato starch (INCI: Potato starch, PPZ Trzemeszno Trzemeszono, Poland), regular rice starch (INCI: Rice starch, Beneo, Brussels, Belgium), tapioca starch (INCI: Tapioca starch, Thai Pride, Thailand, corn starch (INCI: Corn starch) and waxy maize starch (INCI: Waxy maize starch), both supplied by Cargil Poland, Warsaw. Based on these ingredients, a waterless cocoa butter-based lotion bar was formulated. The detailed recipe of the bars is presented in Section 2.3.

Despite their different botanical origins, all starches used in this study are approved as cosmetic raw materials. They differ in particle size and shape. Potato starch consists of large, elliptical granules. Corn and waxy corn starch have medium-sized granules with spherical and polyhedral shapes, respectively. Tapioca starch has small, polygonal granules. Rice starch features small, polyhedral granules. These starches also vary in amylose content, with the highest found in potato starch (20–30%) and the lowest in tapioca starch (approximately 15%). These structural characteristics influence their swelling and gelling abilities as well as the stability and sensory properties of the final cosmetic formulations [19].

### 2.2. Characterization of Starches

#### 2.2.1. Morphology

Microscopic observations of starch were conducted using a Static Image Analysis—Morphologi G3 optical microscope from Malvern (Malvern, UK). The measuring system was equipped with a Nikon CFI 60 optical setup, a high-resolution CCD digital camera, and a computer. By selecting the appropriate magnification, it was possible to analyze particles ranging from 0.5 to 1000 μm in size. Prior to a measurement process, a Standard Operating Procedure (SOP) must be established, defining all measurement settings to ensure the reproducibility of the parameters. During the experiments, the following parameters were determined: the number of particles, the minimum and maximum diameters, equivalent diameter, and the D_10_, D_50_, D_90_, and D_43_ values. D_10_, D_50_, and D_90_ represent the particle diameters at 10%, 50%, and 90% of the cumulative volume distribution, respectively. D_43_ is the volume-weighted mean diameter (De Brouckere mean) that emphasizes larger particles in the particle size distribution. The size of irregular particles is also represented by the Circular Equivalent Diameter (CE diameter). This is defined as the diameter of a circle with the same surface area as the perpendicular projection of the particle’s contours onto the substrate on which it rests in its most stable position. The CE diameter is determined according to the following Equation (1):

(1) $$d_{CE}=\frac{\surd 4A}{\pi }$$

where (A) is the area of the particle, referred to as the “throw area”.

Circularity is defined as the ratio of the circumference of a circle of the same area to the circumference of the particle image. A perfect circle has a circularity of 1, while irregular or spiky particles have a value close to zero. Convexity is a quantitative metric used to measure the surface roughness of a particle. It is calculated by dividing the convex hull perimeter by the perimeter of the particle itself. A smooth shape has a convexity of 1, while a very spiky or irregular object has a convexity closer to 0. Elongation is defined as the difference between unity and the ratio of width to length. Shapes that are symmetrical about all axes have an elongation close to 0, while needle-type and stick shapes have an elongation close to 1. It is important to note that elongation does not characterize roughness [20].

The measurements were repeated three times, each time using a fresh portion of starch from the same production batch.

#### 2.2.2. Water and Oil Absorption Capacities

To determine the water and oil absorption capacities of the starches used, a slightly modified version of the method proposed by Thanyapanich et al. [1] was applied. To assess water and oil absorption, 5 cm^3^ of distilled water or sunflower oil was added to 0.500 g of each starch sample. The mixtures were allowed to stand at room temperature for 30 min, then centrifuged at 3500 rpm for 30 min. The supernatant was decanted into a graduated cylinder and weighed on an analytical balance. The amount of the absorbed oil or water was calculated by subtracting the weight of the recovered supernatant from the initial weight of the added liquid. The results were expressed as a percentage of water or oil absorbed relative to the initial amount. All measurements were done in triplicate using starch taken from a single production batch.

#### 2.2.3. Moisture Content

The moisture content of the starch samples was determined using an Ohaus MB23PL moisture analyzer (OHAUS Europe GmbH, Nänikon, Switzerland). One gram of each starch sample was placed into the moisture analyzer and heated at 100 °C for 1 min. The percentage of moisture content was then recorded. Experiments were repeated three times with fresh starch portions from the same batch.

#### 2.2.4. Flowability

Bulk Density

Fifty grams of the analyzed starch samples were poured through a glass funnel into a cylinder (V = 100 mL). Bulk density was calculated using Equation (2).

(2) $$\rho_{b}=\frac{M_{s}}{V_{p}}$$

where

ρ_b_—bulk density g/cm^3^
V_p_—the volume occupied by the sample, cm^3^
M_s_—the mass of starch sample, 50 g.

Tapped density

To calculate the tapped density, the starch powders were tapped repeatedly until no further change in volume was observed, as described by Equation (3).

(3) $$\rho_{t}=\frac{M_{s}}{V_{t}}$$

where

ρ_t_—tapped density, g/cm^3^
M_s_—the mass of starch sample, 50 g
V_t_—volume occupied by sample after tapping, cm^3^.

The Hausner ratio is a measure of the flowability of a granular material and is calculated as the ratio of the tapped density to the bulk density.

The Compressibility index (CI), also known as Carr Index, quantifies the tendency of a powder to consolidate. It is expressed as a percentage and calculated using Equation (4).

(4) $$\mathrm{CI}=\frac{100(V_{p}-V_{t})}{Vp}$$

where

V_p_—the volume occupied by the sample, cm^3^
V_t_—volume occupied by sample after tapping, cm^3^.
Measurements were performed in triplicate using starch from the same batch.

### 2.3. Recipe of Body Cocoa Butter Bars and Their Preparation

The components of the oil phase (cocoa butter, beeswax, and sunflower oil) were weighted and heated to 80 °C to obtain a homogeneous, clear mixture. Subsequently, the pre-weighed starch was gradually added and the mixture was stirred using an IKA Stirrer (IKA-Werke GmbH & Co. KG, Staufen, Germany) at 300 rpm for 5 min while maintaining the temperature. Afterward, the heating was discontinued and stirring continued until the mass reached a temperature of 40 °C. At this point, vitamin E was added to the system and thoroughly mixed. The warm mass was poured into molds and left to solidify at room temperature for 24 h. After this period, the obtained bars were removed from the molds and evaluated. According to data described in the literature [21], starch finds its applicability as a binder in cosmetics and pharmaceutical formulations at concentrations of 5–15%. Due to the high content of lipid substances susceptible to oxidation or rancidity, tocopheryl acetate was added to the formulation as an antioxidant.

The general recipe of the manufactured anhydrous lotion body bars is presented in Table 1.

### 2.4. Physicochemical Characterization of Products

#### Visual Assessment, Functional Properties, and Stability of Products

Twenty-four hours after pouring the cosmetic mass into the silicone mold, the body bars (with and without starch) were demolded and their external appearance was evaluated. The assessment included shape, color, odor, uniformity (homogeneity), and ease of removal from the mold. The ease of removal was evaluated by manually demolding the body bar. A sample was classified as easy to demold if it could be removed intact, without damage, and without the aid of a spatula.

Products that retained a uniform, bar-like shape after demolding were subjected to stability testing. Samples were stored at 25 °C and wrapped in cellulose paper to prevent fat leakage. After one week, three weeks, and one and three months, the color, shape, smell, and uniformity were reassessed.

The analysis of functional properties included the evaluation of the product’s suitability as a body lotion bar and the sensory impressions during and after skin application (Table 2). This assessment was performed by a qualified evaluator as part of routine quality control. The evaluator had laboratory experience in cosmetic quality control, including routine assessments of sensory parameters such as appearance, color, odor, and texture. In addition, the evaluator had undergone practical training in identifying deviations from standard cosmetic formulation attributes.

Sensory characteristics were assessed descriptively by a single qualified evaluator.

Although sensory panels are typically used for consumer-oriented studies, in this case, the evaluation was limited to verifying the presence or absence of expected formulation features under laboratory conditions. This approach minimized inter-evaluator variability and was considered sufficient for the preliminary formulation screening performed in this study.

The study was conducted under laboratory conditions with controlled temperature, relative humidity, and adequate lighting conditions. All of the tested preparations were evaluated at the same temperature of 21 ± 0.5 °C.

At this stage of the study, three independent series of body bars were manufactured and tested.

All of the body lotion bars that remained stable for three months were also subjected to rheological properties testing and texture analysis.

### 2.5. Rheological Properties

Flow and apparent viscosity curves were determined using the C25-1 cone-plate system with a Brookfield RS Plus rotational rheometer (LaboPlus, Warsaw, Poland). The shear rate range was 1–500 s^−1^, with a measurement time of 60 s corresponding to 60 measurement points. Measurements were performed at 25 °C and 32 °C (Huber Ministat 125 thermostat, Warsaw, Poland) in triplicate. A temperature of 32 °C was chosen to simulate the surface temperature of human skin.

The rheological properties were described using the De Kee model (5):

(5) $$\eta_{app}\left(\dot{\gamma }\right)=\tau_{0}\cdot {\dot{\gamma }}^{-1}+\eta_{1}\cdot \exp\left(-t_{1}\cdot \dot{\gamma }\right)+\eta_{2}\cdot \exp\left(-t_{2}\cdot \dot{\gamma }\right)$$

The estimated parameters were yield stress $\tau_{0}$ (Pa), the exponents $t_{p}$ with a time dimension, and coefficients $\eta_{p}$, *p* = 1.2 with the dimension of the viscosity. Coefficient values of $\eta_{p}$ described the intensity of the time constants $t_{p}$. The estimation of parameters was according to the Marquardt–Levenberg method, which was applied as the minimization algorithm using the least squares method. The target function is defined as follows (6):

(6) $$\chi_{M-L}^{2}=\sum_{j=1}^{N}{\left[\eta_{j}^{\sigma }-{\hat{\eta }}_{j}({\dot{\gamma }}_{j})\;\right]}^{2}\underset{\tau_{0},\;\eta_{1},\eta_{2},t_{1},t_{2},}{\to }\;min$$

where $\eta_{j}^{\sigma }$ is the experimental value of apparent viscosity and ${\hat{\eta }}_{j}$ was calculated from the De Kee equation.

### 2.6. Texture Analysis

Testing the hardness of the resulting body bars was carried out using a SHIMAZU EZ Test EZ-LX texturometer machine (Shimazu Scientific Instruments, Kyoto, Japan) equipped with a needle-shaped probe (ϕ = 2 mm). A penetration test was performed in two variants, developed on the basis of ASTM D1321-10 [23]. In the first variant, penetration was carried out to a depth of 5 mm, while in the second, penetration was carried out until the lotion bar was completely penetrated (breakthrough). In both variants, a test speed of 1 mm·s^−1^ was applied. Measurements were performed at 25 °C in five repetitions for each bar type.

### 2.7. Statistical Analysis

Tests of the water and oil absorption capacities, moisture contents, and flowability properties of starches were performed in at least triplicate and expressed as mean ± standard deviation. One-way analysis of variance (ANOVA) was used to evaluate differences among the samples. The assumption of normality was verified using the Shapiro–Wilk test, and the data met the requirements for ANOVA. No statistical or visual evidence of significant outliers was observed. A significance level of *p* = 0.05 was applied. For statistically significant results, Tukey’s HSD post hoc tests were used for multiple comparisons. Lowercase letters indicate statistically significant differences; samples labeled with different letters differ significantly.

## 3. Results

### 3.1. Characterization of Starches

#### 3.1.1. Morphology

**Figure 1:**
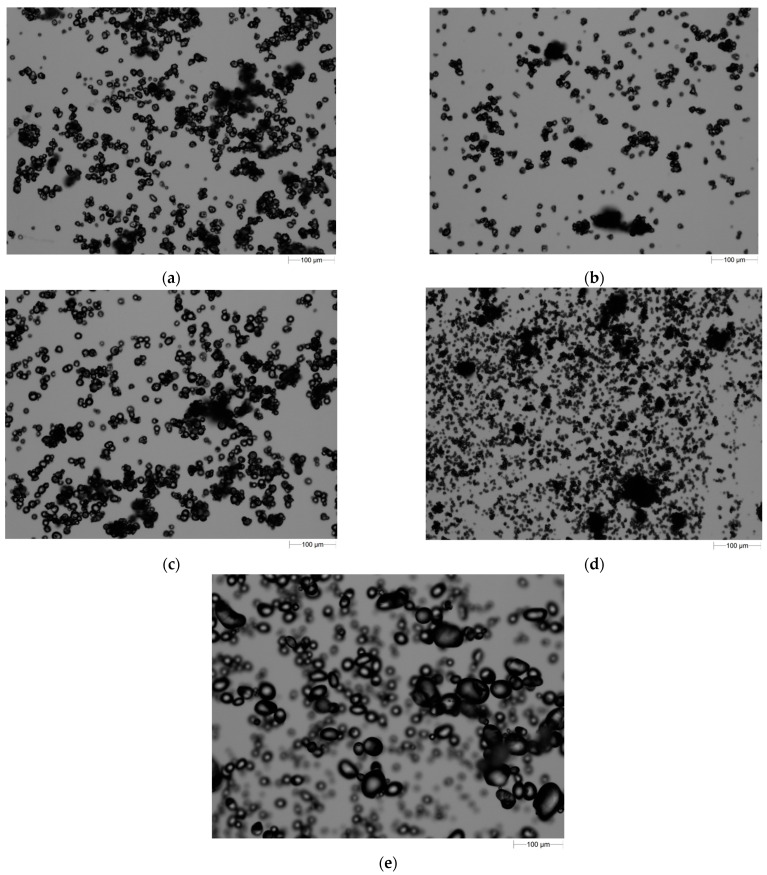
Microscopic images of starches used in the study (**a**) corn starch; (**b**) waxy starch; (**c**) tapioca starch; (**d**) rice starch; (**e**) potato starch.

**Figure 2:**
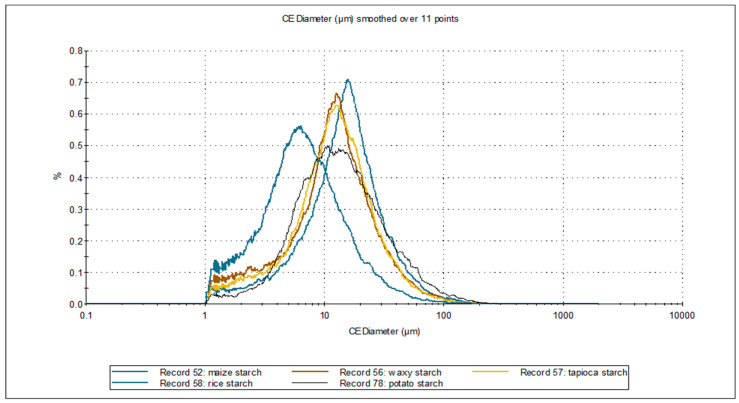
CE diameter number distribution of investigated starches.

Starches isolated from different botanical sources exhibit characteristic granular morphologies; therefore, microscopic analysis allows the determination of the average granule size and botanical origin. The morphological characteristics of starches, including particle size and shape parameters obtained through static image analysis, are presented in Table 3. Microscopic images and the CE diameter number distributions of the investigated starches are illustrated in Figure 1 and Figure 2.

The equivalent diameter of the analyzed starches ranged from 9.31 to 20.01 µm. The smallest diameter was recorded for rice starch; the maximum particle diameter, below which 90% of the sample volume occurs (D_90_), was 17.75 µm. The remaining starches exhibited similar equivalent diameters. The D_50_ diameter, also known as the median of the distribution—indicating the particle size at which 50% of the sample volume is smaller and 50% is larger—was the smallest for rice starch (6.48 µm). For the other starches, the values were several micrometers. Microscopic images (Figure 1) reveal a tendency of starch particles to form agglomerates. Additionally, particle adhesion and overlapping were observed. This is supported by the value of the maximum diameter, which ranged from 275.5 to 337.2 µm, and the average De Brouckere diameter (D_43_), which ranged from 100.53 µm for tapioca starch to 143.1 µm for rice starch. Potato starch showed the highest circularity (0.760) and the lowest elongation (0.254). In contrast, waxy maize starch was characterized by the lowest circular shape (0.630), indicating a more elongated shape with the highest elongation value (0.301). The smallest variation among the morphological parameters was recorded in convexity, with values ranging from 0.902 to 0.954. It should be emphasized that for circularity, elongation, and convexity, no statistically significant differences were found for the analyzed types of starch.

#### 3.1.2. Water and Oil Absorption Capacities, Moisture Content, and Flowability

Table 4 shows the moisture content as well as the percentage of water and oil absorbed by the various types of starch used in the tests.

The samples analyzed were characterized by low moisture content, ranging from 2.50 to 5.47%. The highest moisture content was found in potato starch, while the lowest was recorded for rice starch. The remaining starches had moisture content at a similar level, with no statistically significant differences observed. Additionally, all of the analyzed starches showed a higher percentage of oil absorption compared to water, ranging from 22.9 to 28.3% for oil and from 12.4 to 16.4% for water. Rice, corn, and tapioca starches exhibited similar water absorption capacities (approximately 15–16%), which were higher than those of waxy and potato starches, which absorbed approximately 3–4% less water. Rice and tapioca starch had the highest oil absorption capacities. Potato and waxy starches showed lower oil absorption capacities. The oil absorption percentage of corn starch did not differ statistically from either of these two groups. Potato starch showed the highest bulk density, whereas rice starch had the lowest. The bulk density of tapioca starch did not differ statistically from those of the corn and waxy starches. The Hausner ratio values, indicating flowability, ranged from 1.25 for potato starch to 1.66 for rice starch.

### 3.2. Physicochemical Properties

#### Visual Assessment, Functional Properties, and Stability of Obtained Body Bars

**Figure 3:**
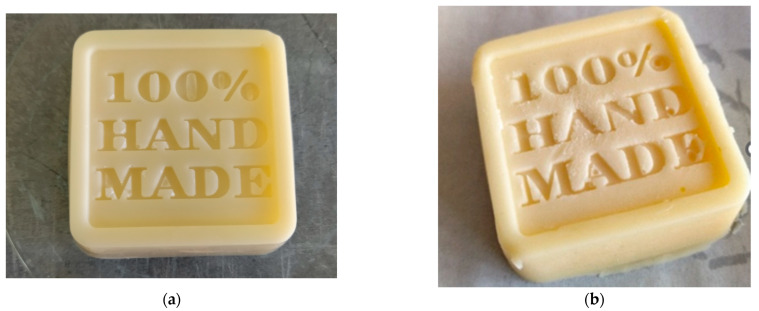

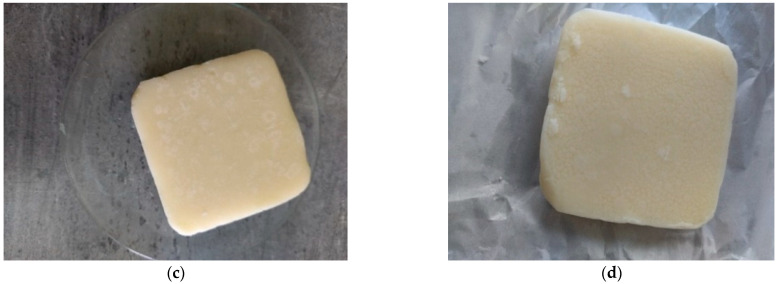
Photos of obtained lotion body bars: (**a**) stable bar with 5% potato starch content; (**b**) signs of non-homogeneity in a bar with 5% rice starch content; (**c**,**d**) signs of instability in bars observed during storage.

Table 5 presents the results of visual assessment of the obtained body bars. Products in the form of cubes which could be introduced on the market were subjected to stability tests. The results of the stability tests are presented in Table 6 and Figure 3.

A decrease in beeswax content in the formulation, replaced by starch, reduces the stability of the samples. However, depending on the type of starch used, it is impossible to obtain a bar in a form suitable for commercial use. The maximum starch concentration at which the samples remained stable for three months was 5.0%. At this level, only potato, tapioca, and waxy starches could be used; for rice and corn starch, the maximum allowable concentration in the recipe was 2.5%. Samples containing 7.5% starch were stable for up to one month. At a concentration of 10% (*w*/*w*), only the formulation with potato starch could be obtained; however, it lacked suitable application properties, as it was too soft and difficult to remove from the mold. No stable formulations were obtained with a 15% (*w*/*w*) starch content.

The most commonly observed signs of instability included fatty exudation and starch sedimentation at the bottom of the lotion body bar (Figure 3b). During storage, the bars hardened and crumbled, and the oil leaked onto the paper in which they were stored (Figure 3c,d). The results of the functional properties of the obtained lotion body bars are presented in Table 7.

### 3.3. Rheological Analysis

**Figure 4:**
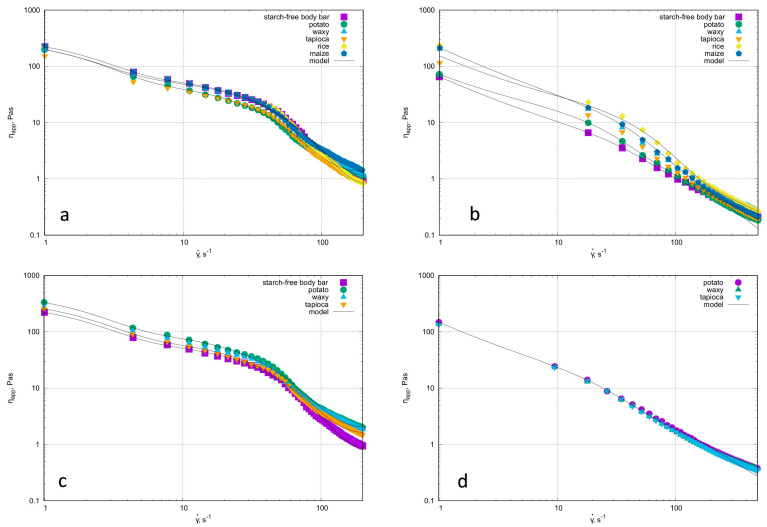
Dependence of apparent viscosity on shear rate for cosmetic formulations without (starch-free body bar) and with starch concentrations of 2.5% at 25 °C (**a**) and 32 °C (**b**) and 5.0% at 25 °C (**c**) and 32 °C (**d**).

All of the tested systems, regardless of the temperature at which the rheological properties were measured, behaved as shear-thinning and deviated from the exponential rheological model (Figure 4). The presence of a maximum on the flow curve (indicates the yield stress phenomenon. In the range of lowest shear rates (<10 s^−1^), the apparent viscosity values were practically independent of both the starch concentration and the botanical origin of the starch in the formulation. An increase in temperature up to 32 °C (a close to the skin application temperature of the formulation) results in a decrease in the apparent viscosity range. It is worth noting that the values of this parameter under shear rate conditions of 1 s^−1^ were greater than 100 Pas and did not depend on either starch concentration or temperature. For formulations containing 5.0% rice or maize starch, reliable rheological measurements could not be obtained due to the unstable nature of the samples. Analysis of the shear rate dependence of apparent viscosity indicated a stabilizing effect of starch in the tested formulations. At 25 °C, for all preparations, including the one without starch, the apparent viscosity’s dependence on shear rate were similar in value and independent of starch concentration (Figure 4a,c) or starch type. The rheological behavior of formulations including 2.5% starch at higher temperatures (Figure 4b,d) suggests that the addition of starch at this level stabilized the system. At higher concentrations (5.0%), the botanical origin of the starch did not influence the $\eta_{app}\left(\dot{\gamma }\right)$ relationship.

A DeKee model with a yield stress was used to describe the rheological properties (Table 8).

Analysis of the estimated values of parameters from the De Kee equation of state showed no differences in the values of the time constants $t_{1}$ and $t_{2}$ as a function of starch concentration and type. The noticeable differences were in the intensities of the time constants represented by $\eta_{1}$ and $\eta_{2}$ as a function of starch concentration, but this has its origin in the course (values) of the apparent viscosity. For lower (2.5%) starch concentrations (except for potato starch), viscosity effects predominated, as evidenced by the higher intensity of time $t_{1}$ (longer characteristic time, responsible for viscosity effects). In the case of potato starch, the different behavior may be determined by the fact that it is an ionic starch—a natural phosphate [24]. However, differences can be seen in the values of the yield stress ($\tau_{0}$) at 25 °C. For potato starch at the lower concentration of 2.5%, the yield stress was slightly lower than it was for the 5% concentration. For waxy and tapioca starch, $\tau_{0}$ at a concentration of 2.5% was practically two times lower than at 5%. An increase in temperature resulted in an increase in the yield stress and a decrease in the time constants, with the exception of the potato starch bars. This may be due to the ionic nature of this starch—the starch granules interact differently with the fatty phase and offer greater resistance to deformation. An increase in temperature unifies the rheological properties of the systems studied: the range of apparent viscosity does not change, regardless of temperature, being in the range 0.1 Pas to ~300 Pas. It can be concluded that the addition of starch stabilizes the tested suspensions in this respect. Increasing the starch addition to 5% in the cases of potato, waxy, and tapioca starch resulted in a complete unification of the rheological properties.

### 3.4. Texture Analysis

The texture parameters of the tested systems are shown in Table 9. Missing values for some systems (corn and rice 5.0%) indicated that they were not tested due to their low stability during storage (Figure 3). The hardness of the starch-free body bar (comparison sample) was the highest of the systems tested at 3.636 N. The values of the systems containing 2.5% starch ranged from 3.215 N to 3.462 N. The use of 2.5% starch resulted in a reduction in the hardness of the body bars compared to the starch-free body bar. The lowest value was recorded for the system containing 2.5% added potato starch and the highest value for waxy maize starch. No significant differences with respect to the starch-free body bar were recorded for the 2.5% waxy sample. Breakthrough of the samples was achieved at a lower force value than in the starch-free body bar. Increasing the proportion of starch in the test systems to 5% also resulted in a reduction in the hardness of the systems with respect to the starch-free body bar. The instability of the two previously mentioned types of bars was also noted. The hardness of the stable systems ranged from 2.895 N to 3.461 N for waxy and tapioca 5.0%, respectively. No significant differences with respect to the starch-free body bar were recorded for the 5.0% tapioca sample. Breakthrough of the tested systems also occurred at a lower force value than for the starch-free body bar. The texture parameter values of the body bars indicate that starch incorporation reduces both hardness and the force required to fracture the bar. This means that reducing the wax content in favor of starch results in bars with a softer texture than those obtained without its addition. Starch, therefore, despite having a binding function like wax, causes body bars to have altered textural properties.

## 4. Discussion

### 4.1. Selection of Ingredients for Anhydrous Lotion Body Bars

Body bars are composed of natural ingredients that remain solid at room temperature but melt upon contact with body heat, which corresponds to a skin surface temperature of approximately ~32–34 °C. Due to the properties of the main ingredient—cocoa butter—the formulation is prone to forming slowly solidifying polymorphic forms, which may affect the product’s stability. This tendency was particularly noticeable in samples containing 5% rice and corn starch (Table 5 and Figure 3). During storage, a fat bloom also appeared, which is an unfavorable phenomenon occurring in cocoa butter that results from temperature changes and may indicate a lower quality of the raw material used [25].

However, in anhydrous solid formulations such as body lotion bars, vegetable butter alone does not provide sufficient hardness for the product to maintain its shape and be suitable for consumer use. Therefore, waxes are added to the recipe to enhance structural integrity. The harder the butter used, the less wax is required—cocoa butter, for instance, allows for minimal wax usage [26]. Beeswax, classified as soft wax due to its relatively low melting point (62 °C), contributes to achieving a solid product with the proper degree of flexibility. Waxes also form an occlusive layer on the skin, thereby reducing transepidermal water loss (TEWL). Within this structural framework, starch can be incorporated into the formulation. As shown by the results of the visual assessment (Table 5), stability (Table 6), and texture profile analysis (Table 9), starch influences the functional characteristics of the product. However, achieving the desired hardness and form stability still depends primarily on the presence of wax in the formulation.

The type and amount of vegetable oil used determines how quickly the cosmetic product is absorbed and spread on the skin. To ensure that the lotion body bar spreads well on the skin, at least one-third of the formulation should consist of liquid oils [26]. Heavy oils should be avoided in favor of light oils (from the drying or semi-drying group), such as sunflower oil, which was used in these formulations.

Anhydrous lotion body bars provide an intermediate moisturizing effect by leaving a thin hydrophobic layer on the skin after application. This layer is absorbed gradually, leaving a noticeable oily or greasy sensation. As previously mentioned, the greasiness of these preparations can be achieved by using the appropriate vegetable oil, as well as by adding fatty alcohols to the formula, which improve the glide during application and enhance the consistency of the bar. In addition, the inclusion of powders such as starch can contribute to a skin feel [26].

### 4.2. The Impact of Starch on the Characteristics of Obtained Products

Starches from different botanical origins vary in their physicochemical properties and related technological suitability. One of the important tests relevant both to processing and the quality of the final product is the moisture content in the raw material. In the present study, the moisture levels determined in the analyzed starches were lower than those reported in the literature. For potato starch, the moisture content typically ranges from 15.8 to 19.0% [27,28]. For rice, the reported value is approximately 8.01% [28]. Corn and waxy starches contain 9.5% and 9.0–13% moisture, respectively [29], and for tapioca the level is 10–13.5% [30].

These differences may result from production technology and manufacturer specifications, as in Poland starches that are additionally dried before being placed on the market are commonly available. The water and oil absorption capacities of starch are attributed to the presence of hydrophilic and hydrophobic groups, respectively, as well as to the pores and cracks present in starch granules [31]. This variation may be attributed to differences in protein concentrations, the extent of interactions with water and oil, and possibly their conformational characteristics. The lower water absorption capacity observed in analyzed samples may result from a reduced availability of polar amino acids. Hydrophobic proteins present in starch exhibit a high affinity for lipids, binding oil both physically through capillary attraction and chemically through the interaction of non-polar amino acid side chains with the paraffin chains of fats. The presence of lipids in starch (usually enclosed inside the amylose helix) hinders the swelling of grains [32,33,34].

According to Hibu Odi et al. [35], starch with good oil absorption capacity can be used as an oil retention agent. It can also reduce the greasiness of the skin after applying the product. The ability of starch to absorb oil is also a measure of the emulsifying potentials of the starch [33].

The starches used in the bar formulation absorbed excess oil at a capacity exceeding 20%, thereby reducing the greasiness of the products without causing a grainy texture. This was confirmed by the results of sensory analysis for the products obtained (Table 5, Table 7). All of the lotion body bars had an aesthetically pleasing appearance and a pleasant fragrance. The samples were non-sticky and spread very well on the skin. Compared to both the starch-free system and the systems formulated with 5% starch, the bars containing 2.5% starch showed a noticeable reduction in residual greasiness after application. In waterless products such as body bars, it is therefore important that the raw material (starch) used has a low moisture content and a high oil absorption capacity.

According to A.M. Ricón et al. [4] the performance properties of granular starches can be dependent on such variables as particle size and shape. Very fine particles (5 µm) will have problems with caking and stickiness. Larger particles (15–20 µm) have improved flow properties. A truncated shape, like tapioca starch, will have a more cushiony and less silky feel than other starches with similar sizes but different shapes, such as corn starch.

Furthermore, from a functional standpoint, an important parameter for body bars is the convexity of the starch. Convexity is a parameter that describes the particle’s roughness and smoothness. Values closer to 1 indicate smoother particles. The convexity values above 0.900 for the analyzed starches suggest that these particles should not cause an unpleasant sensation on the skin. However, a scratching or rough feeling was observed for rice starch, which had the smallest D_50_ diameter among the starches tested. In our opinion, this may be related to differences in flowability.

A comparison of the rheological properties of bars containing 2.5% starch for all of the tested starches may indicate that the equivalent diameter may influence the yield stress: the value of $\tau_{0}$ increased with the increasing D_CE_, except in the case of tapioca starch. A similar trend was observed at 32 °C, with the exception of the potato starch system, which exhibited a notably low yield stress. An analysis of the relationship between bulk density and yield stress at 25 °C showed that at the lowest starch concentration (2.5%), a lower bulk density generally corresponded to a lower yield stress, again with the exception of tapioca starch. At 32 °C, potato starch, which had the highest bulk density among the starches tested, does not follow this trend.

According to the European Pharmacopeia, the Hausner ratio indicates that only potato starch exhibits good flow properties, tapioca starch has fair flow properties, and the remaining starches have poor flow abilities. Solid raw materials with good flowability mix more uniformly, ensuring the homogeneity of the blend, whereas powders with poor flowability are prone to segregation. Starches with low flowability tend to sediment and exhibit stronger interparticle interactions compared to materials with good flowability. This is confirmed by the large differences observed between bulk density and tapped density, as well as by instability phenomena such as sedimentation of starch within the bar (Table 5). The interactions of particles are also evident in the flow curves.

Observed instability phenomena, such as sedimentation of starch particles within the bar matrix (Table 5), are influenced by the density difference between the starch and the surrounding lipid phase [36]. According to general sedimentation theory, particle size and shape also play important roles. A significant density mismatch between dispersed particles and the continuous phase, low viscosity, and large particle size promote high sedimentation velocities.

As noted by Cretella et al. [36], the hydration process of starch particles may also affect their sedimentation behavior. In the present study, the starches used exhibited higher capacities for oil absorption than for water absorption, and they were incorporated into an anhydrous system. Initially, the system was in a liquid state and then gradually transitioned into a solid matrix upon cooling. This transformation likely affected particle mobility and distribution within the bar, with sedimentation occurring primarily during the liquid phase prior to solidification.

Oil absorption capacity (OAC) influences the yield stress ($\tau_{0})\;$of the tested systems. At 25 °C, $\tau_{0}$ decreases with an increasing OAC, whereas at 32 °C a maximum of $\tau_{0}$ was observed as a function of OAC (notably for maize starch). At 25 °C, samples with higher oil absorption capacities exhibited lower yield stress, likely due to increased internal lubrication and reduced structural rigidity. This suggests that OAC may facilitate better oil distribution within a matrix, decreasing intermolecular friction and enhancing product spreadability. However, at 32 °C—close to body temperature—the partial melting of the fat phase (comprising 50% cocoa butter by mass) likely alters starch-lipid interactions. This transition may promote oil migration from the matrix to the surface, resulting in microstructural rearrangement. Additionally, changes in surface texture and molecular mobility at higher temperatures may modify the contact area and flow behavior, contributing to the observed reversal in the OAC–yield stress relationship.

## 5. Conclusions

The results of this study contribute to a better understanding of the impact of starches from various botanical origins on the functional and mechanical properties of anhydrous lotion body bars. The botanical origin of the starches did not significantly affect the rheological properties of the anhydrous lotion bars. However, the presence of starch itself influenced the rheological profile, with apparent viscosity values (1–300 Pa·s) remaining consistent across all tested types at both 25 °C and 32 °C. All bars—including the starch-free body bar—maintained acceptable textural integrity and usability. The addition of starch powders with a low moisture content and high oil absorption capacity may enhance spreadability and reduce greasiness. Based on apparent viscosity and ease of application, a 2.5% starch concentration appears to be optimal.

## Figures and Tables

**Table 1:** General recipe of the manufactured body bars along with the function of individual ingredients.

Ingredients	% Mass.							Function
Cocoa butter	50.0	50.0	50.0	50.0	50.0	50.0	50.0	Skin conditioning—emollient, moisturizing effect, softens the skin, improves its elasticity [22]
Sunflower oil	34.0	34.0	34.0	34.0	34.0	34.0	34.0	Skin conditioning—emollient, nourishes the skin, softens and moisturizes, may contain antioxidants that slow down the effects of aging [22]
Beeswax	0	2.5	5.0	7.5	10.0	12.5	15.0	Binding, skin conditioning, viscosity controlling, consistency-forming substance [22]
Starch	15.0	12.5	10.0	7.5	5.0	2.5	0	Binding, viscosity controlling, thickening agent, absorbs excess oil, soothing, protecting the skin [22]
Vitamin E (tocopheryl acetate)	1.0	1.0	1.0	1.0	1.0	1.0	1.0	Antioxidant [22]

**Table 2:** Description of attributes evaluated in functional properties analysis, by category.

Phase	Sensory Attribute	Definition	Descriptive Terms
Before rubbing	Appearance	First impression of the consumer upon viewing the product.	Pretty, nice, ugly
	Odor	The sensory impression caused by interaction of volatile chemical compounds and olfactory receptor cells.	Extremely nice, very nice, nice, neutral, unpleasant, unsavory
During pick-up (Attributes measured by manipulation between fingers)	Consistency	It is the result of the density and cohesiveness of the formulation.	Liquid, semisolid, solid
During rubbing (Attributes measured by rubbing the product on the skin)	Initial sensations upon contact with skin	Impressions upon applying the sample to the skin	Extremely nice, very nice, nice, neutral, unpleasant
	Spreadability	Ease of spreading the product on the skin	Very good, optimal, satisfactory, bad, very bad
	Stickiness	Degree of stickiness of the sample—the force required to separate the finger from the skin.	Not sticky, slightly sticky, sticky, very sticky
After feel (Attributes of the skin surface after the use of product)	Residual greasiness after application(oiliness)	Degree of greasy deposits on the skin immediately after application (greasiness) and after approximately 30 min (lubrication).	Low, optimum, high, too high

**Table 3:** Morphological parameters of tested starches—Static Image Analysis.

Type of Starch	Number of Particles	D_CE_ [μm]	D_min_ [μm]	D_max_ [μm]	D_10_ [μm]	D_50_ [μm]	D_90_ [μm]	D_43_ [μm]	Circularity	Elongation	Convexity
Corn	35,609 ± 293 a	18.28 ± 0.46 ab	1.45 ± 0.51 a	319.72 ± 39.57 a	4.80 ± 0.13 ab	13.71 ± 1.46 a	34.77 ± 0.23 b	132.6 ± 20.03 ab	0.731 ± 0.04 a	0.281 ± 0.01 a	0.946 ± 0.01 a
Potato	35,592 ± 64 a	20.01 ± 0.82 a	1.09 ± 0.00 a	337.2 ± 25.05 a	5.12 ± 0.44 a	13.49 ± 0.92 a	41.18 ± 1.25 a	130.3 ± 4.0 ab	0.760 ± 0.00 a	0.254 ± 0.01 a	0.954 ± 0.00 a
Waxy	35,407 ± 199 a	15.39 ± 0.36 c	1.09 ± 0.00 a	318.7 ± 40.35 a	2.97 ± 0.20 bc	11.62 ± 0.49 a	29.75 ± 0.27 c	118.2 ± 4.28 ab	0.630 ± 0.00 a	0.301 ± 0.00 a	0.902 ± 0.00 a
Rice	35,668 ± 137 a	9.31 ± 0.05 d	1.09 ± 0.0 a	306.75 ± 1.35 a	2.42 ± 0.24 c	6.48 ± 0.15 b	17.75 ± 0.31 a	143.1 ± 5.89 a	0.696 ± 0.09 a	0.299 ± 0.02 a	0.938 ± 0.02 a
Tapioca	35,648 ± 300 a	17.25 ± 1.60 bc	1.09 ± 0.0 a	275.53 ± 10.31 a	5.20 ± 1.43 ab	13.42 ± 1.35 a	31.44 ± 2.11 bc	100.53 ± 7.10 b	0.700 ± 0.08 a	0.295 ± 0.02 a	0.934 ± 0.02 a

D_CE_—Equivalent diameter, D_min_—Minimum diameter, D_max_—Maximum diameter, D_10_, D_50_, and D_90_ represent the particle diameters at 10%, 50%, and 90% of the cumulative volume distribution, respectively. Samples with the same letter are not significantly different. Samples labeled with a combination of letters (e.g., “ab”) are not significantly different from those marked with the letters “a” and “b”.

**Table 4:** Moisture content, percentage of water and oil absorbed by various types of starch.

Type of Starch	Water Absorption [%]	Oil Absorption [%]	Moisture Content [%]	Bulk Density [kg/m^3^]	Tapped Density [kg/m^3^]	CI	H Ratio	Flow Characteristics
Potato	13.1 ± 0.8 b	22.9 ± 1.2 b	5.47 ± 0.09 a	744.02 ± 27.9 a	934.2 ± 39.7 a	25.4	1.25	Good flow
Waxy	12.4 ± 0.4 b	23.8 ± 1.3 b	4.21 ± 0.37 b	471.4 ± 4.67 c	685.8 ± 13.53 c	45.4	1.45	Poor flow
Rice	16.0 ± 0.6 a	28.3 ± 0.3 a	2.50 ± 0.20 c	400.6 ± 5.38 d	665.3 ± 22.52 c	65.9	1.66	Poor flow
Corn	16.4 ± 0.4 a	25.7 ± 0.7 ab	3.83 ± 0.31 b	521.5 ± 9.19 b	762.5 ± 14.63 b	46.1	1.46	Poor flow
Tapioca	15.1 ± 0.2 a	26.5 ± 0.8 a	4.36 ± 0.12 b	507.6 ± 5.14 bc	705.5 ± 7.85 bc	38.9	1.38	Fair flow

Samples with the same letter are not significantly different. Samples labeled with a combination of letters (e.g., “abc”) are not significantly different from those marked with the letters “a” “b” and “c”.

**Table 5:** Visual assessment and functional properties of cocoa butter body bars.

Type of Starch	Concentration of Starch [%]			
	2.5	5.0	7.5	10.0
Potato starch	Cube-shaped, light yellow, odor of cocoa butter, homogeneous, easy to remove from the mold, suitable hardness for commercial use	Cube-shaped, light yellow, odur of cocoa butter, homogeneous, easy to remove from the mold, suitable hardness for commercial use	Cube-shaped, light yellow, odur of cocoa butter, homogeneous, easy to remove from the mold, suitable hardness for commercial use	Cube-shaped, light yellow, odor of cocoa butter, non-homogeneous, difficult to remove from the mold, too soft
Corn starch	Cube-shaped, light yellow, odor of cocoa butter, homogeneous, easy to remove from mold, suitable hardness for commercial use	Cube-shaped, light yellow, odor of cocoa butter, homogeneous, easy to remove from the mold, suitable hardness for commercial use	Cube-shaped, light yellow, odor of cocoa butter, homogeneous, very soft, difficult to apply, difficult to remove from the mold	−
Rice starch	Cube-shaped, white to yellow, odor of cocoa butter, homogeneous in appearance, slightly scratchy in feel, easy to remove from the mold, suitable for commercial use	Cube-shaped, white to yellow, odor of cocoa butter, non-homogeneous—scratchy, easy to remove from the mold, suitable hardness for commercial use	Cube-shaped, white to yellow, odor of cocoa butter, non-homogenous—scratchy, very soft, impossible to apply to the skin, difficult to remove from the mold	−
Tapioca starch	Cube-shaped, light yellow, odor of cocoa butter, homogeneous, easy to remove from the mold, suitable hardness for commercial use	Cube-shaped, light yellow, odor of cocoa butter, homogeneous, easy to remove from the mold, suitable hardness suitable for commercial use	Cube-shaped, light yellow, odor of cocoa butter, easy to remove from the mold, suitable hardness for commercial use, visible starch clusters, non-homogeneous, rough in contact with the skin	−
Waxy starch	Cube-shaped, light yellow, odor of cocoa butter, homogeneous, easy to remove from the mold, suitable hardness suitable for commercial use	Cube-shaped, light yellow, odor of cocoa butter, homogeneous, easy to remove from the mold, suitable hardness suitable for commercial use	Cube-shaped, light yellow, odor of cocoa butter, easy to remove from the mold, suitable hardness suitable for commercial use, non-homogeneous, visible starch clusters, rough in contact with the skin	–
Starch-free body bar	Cube-shaped, light yellow, odor of cocoa butter, homogeneous, easy to remove from the mold, suitable hardness for commercial use			

−: the studies were discontinued due to the lack of stability and/or a form suitable for skin application and commercialization in a lower concentration system.

**Table 6:** Results of stability tests for obtained body lotion bar.

Storage Period	Starch-Free Body Bar	Potato Starch Concentration [%]			Corn Starch Concentration [%]		Rice Starch Concentration [%]		Waxy Starch Concentration [%]			Tapioca Starch Concentration [%]		
		2.5	5.0	7.5	2.5	5.0	2.5	5.0	2.5	5.0	7.5	2.5	5.0	7.5
One week	+	+	+	+	+	+	+	+	+	+	+	+	+	+
Three weeks	+	+	+	+	+	−	+	−	+	+	+	+	+	−
One month	+	+	+	+	+	−	+	−	+	+	+	+	+	−
Three months	+	+	+	−	+	−	+	−	+	+	-	+	+	−

+ stable product, − unstable product.

**Table 7:** Evaluation of the functional properties of stable cocoa butter-based body bars with and without starch in the range from 2.5 to 5% by mass.

Sensory Attribute	Starch-Free Body Bar	Potato Starch [%]		Tapioca Starch [%]		Waxy Starch [%]		Rice Starch [%]	Corn Starch [%]
		2.5	5.0	2.5	5.0	2.5	5.0	2.5	2.5
Appearance	Pretty	Pretty	Pretty	Pretty	Pretty	Pretty	Pretty	Pretty	Pretty
Odor	Very nice	Very nice	Very nice	Very nice	Very nice	Very nice	Very nice	Very nice	Very nice
Consistency	Solid	Solid	Solid	Solid	Solid	Solid	Solid	Solid	Solid
Initial sensations upon contact with skin	Very nice	Very nice	Very nice	Very nice	Very nice	Very nice	Very nice	Nice	Very nice
Spreadability	Very good	Very good	Very good	Very good	Very good	Very good	Very good	Very good	Very good
Stickiness	Not sticky	Not sticky	Not sticky	Not sticky	Not sticky	Not sticky	Not sticky	Slightly sticky	Not sticky
Residual greasiness after application (oiliness)	Optimum	Low	Optimum	Low	Optimum	Low	Optimum	High	Low

**Table 8:** DeKee model parameters for the tested lotion body bars.

Sample	Temperature [°C]	Concentration of Starch [%]	$\tau_{0}$ [Pa]	$t_{1}$ [s]	$t_{2}$ [s]	$\eta_{1}$ [Pas]	$\eta_{2}$ [Pas]	$\chi_{M-L}^{2}$
Starch-free body bar	25	−	75	0.62	0.03	170	60	0.5
	32	−	60	0.04	0.01	6	0.4	0.3
Potato	25	2.5	133	0.71	0.04	56	37	0.3
		5.0	194	0.80	0.04	136	80	1.6
	32	2.5	56	0.05	0.01	16	1.3	0.2
		5.0	127	0.07	0.01	1	18	0.1
Waxy	25	2.5	93	0.87	0.03	126	54	0.5
		5.0	172	0.64	0.03	45	70	1.6
	32	2.5	184	0.05	0.01	21	0.3	0.2
		5.0	127	0.07	0.01	1	18	0.1
Tapioca	25	2.5	51	0.88	0.04	143	44	0.3
		5.0	154	0.42	0.04	33	59	0.8
	32	2.5	184	0.05	0.01	21	0.3	0.3
		5.0	127	0.07	0.01	1	18	0.1
Rice	25	2.5	57	0.66	0.03	198	66	0.3
	32		132	0.03	0.01	22	0.004	0.5
Maize	25	2.5	111	0.84	0.03	148	58	0.4
	32		198	0.04	0.01	17	0.78	0.3

**Table 9:** Texture parameters of body bars.

Sample	Concentration of Starch [%]	5 mm Penetration Hardness [N]	Breakthrough Force [N]
Starch-free body bar	−	3.636 ± 0.096 a	4.638 ± 0.118 a
Corn	2.5	3.333 ± 0.075 bc	4.180 ± 0.077 bc
	5.0	−	−
Rice	2.5	3.420 ± 0.099 b	4.341 ± 0.118 b
	5.0	−	−
Tapioca	2.5	3.368 ± 0.026 bc	4.293 ± 0.044 bc
	5.0	3.461 ± 0.131 a	3.968 ± 0.157 b
Waxy	2.5	3.462 ± 0.081 ab	4.160 ± 0.115 ab
	5.0	2.895 ± 0.040 b	3.677 ± 0.091 c
Potato	2.5	3.215 ± 0.104 c	3.983 ± 0.095 c
	5.0	3.036 ± 0.082 b	3.574 ± 0.110 c

Samples with the same letter are not significantly different. Samples labeled with a combination of letters (e.g., “abc”) are not significantly different from those marked with the letters “a”, “b” and “c”.

## Data Availability

The original contributions presented in this study are included in the article Further inquiries can be directed to the corresponding author.
